# The safe lowest effective power of subthreshold micropulse laser treatment in Chinese patients with acute or chronic central serous chorioretinopathy

**DOI:** 10.3389/fmed.2024.1494402

**Published:** 2024-11-06

**Authors:** Ting Xie, Wangting Li, Linli Wang, Jiafeng Ning, Zhi Li, Yulei Chen, Xifeng Lin, Shaolin Du, Qingshan Chen

**Affiliations:** ^1^Dongguan Tungwah Hospital, Dongguan, China; ^2^Shenzhen Eye Hospital, Shenzhen Eye Institute, Shenzhen Eye Hospital Affiliated to Jinan University, Shenzhen, China

**Keywords:** central serous chorioretinopathy, subthreshold micropulse laser, central macular thickness, laser power, treatment

## Abstract

**Purpose:**

To assess the safe, lowest effective laser power of subthreshold micropulse laser (SML) for treating acute and chronic central serous chorioretinopathy (CSC) in Chinese patients.

**Methods:**

Patients were distinguished with acute or chronic CSC based on focal or diffuse retinal pigment epithelium (RPE) leakage on fundus fluorescein angiography (FFA), with or without widespread RPE decompensation. Patients were categorized into five groups and treated with 577 nm yellow SML according to 50% titration power. The change of best-corrected visual acuity (BCVA) and central macular thickness (CMT) were set as primary outcomes. A linear regression model assessed the correlation between different factors and outcome indicators.

**Results:**

A total of 103 patients with 127 eyes (61 with acute CSC and 66 with chronic CSC) were enrolled. The baseline characteristics were balanced between the five groups (all *p* > 0.05). The decrease of CMT and the improvement of BCVA were related to the CMT at baseline (all *p* < 0.05). We found that the lowest effective laser power for acute CSC was 425 mW (−225.50 μm vs. −171.24 μm vs. −114.50 μm vs. −130.54 μm vs. −68.00 μm, *p* < 0.001), showing a significant CMT reduction at this power, but no significant increase in BCVA (−0.15 ± 0.10 logMAR vs. −0.20 ± 0.16 logMAR vs. −0.14 ± 0.11 logMAR vs. −0.17 ± 0.30 logMAR vs. −0.11 ± 0.14 logMAR, *p* > 0.05). For chronic CSC, the lowest effective laser power was 375 mW (*p* = 0.01), the change of CMT was significant in 375 mW (−93.91 ± 109.06 μm, −119.32 ± 105.56 μm, −88.67 ± 67.26 μm, −60.89 ± 106.86 μm, and −99.11 ± 157.32 μm, *p* = 0.04). The change of BCVA was similar trend (−0.54 ± 0.66 logMAR vs. −0.17 ± 0.23 logMAR vs. −0.10 ± 0.21 logMAR vs. −0.02 ± 0.30 logMAR vs. 0.05 ± 0.19 logMAR, *p* < 0.001).

**Conclusion:**

In this study, our results suggested 425 mW and 375 mW laser power is the lowest effective SML power for treating acute and chronic CSC in Chinese patients respectively, And the power of SML for chronic CSC requires lower power than acute CSC.

## Introduction

Central serous chorioretinopathy (CSC) is a common chorioretinal disease characterized by an accumulation of subretinal fluid (SRF). The exact pathogenesis is subject to debate, but it was thought to be the serous detachment of the neurosensory retina and the change in the retinal pigment epithelium (RPE) (1, 2). It commonly presents with metamorphopsia, central scotoma, and progressive visual damage. CSC was usually subdivided into acute and chronic CSC. Acute CSC is usually thought to be a self-limited disorder, which resolves within few months with minor permanent damage to vision. In comparison, chronic CSC can lead to clinically significant central vision loss and reduced quality of life (3). However, according to the research reported, the risk of recurrence for acute CSC could be about 50, and 15% might develop persistent SRF, with a duration longer than 4 months would be defined as persistent chronic CSC (4, 5), which could result in irreversible visual loss (6, 7). Therefore, acute CSC need early intervention to accelerate the resolution of SRF, shorten the disease duration and help visual acuity improvement more quickly (8).

Photodynamic therapy (PDT), laser photocoagulation, and intravitreal injection of anti-vascular endothelial growth factor (VEGF) are commonly employed clinical treatments for CSC (9). ICG-guided half-dose PDT has traditionally been regarded as the gold standard for managing CSC. However, due to the high cost and limited availability of verteporfin in China, Subthreshold Micropulse Laser (SML) has emerged as a promising alternative. Unlike conventional lasers that deliver continuous pulses, SML emits energy in a series of micropulses, which reduces the duration of heat conduction and enables repeated micropulse stimulation to promote RPE repair (10). This technique also limits the temperature increase in surrounding tissues, minimizing the risk of significant retinal damage (11, 12).

SML has become a well-established technique in the clinical management of CSC. However, more studies needs to focus on the optimal laser parameters for SML, including exposure time, spot diameter, power, and treatment area selection. While Lijun Zhou et al. have reported differences in efficacy with varying duty cycles (8), the impact of different laser energy levels still needs to be explored. Researches on SML parameters were predominantly led by European and American ophthalmologists (13, 14), with limited studies conducted in China, likely due to the relatively few SML devices available and the challenges in determining optimal treatment settings (9, 15). Additionally, Anastasia V Pilat’s work suggests racial differences in RPE thickness, with Asians exhibiting thicker RPE than Caucasians (16), implying that treatment effects and optimal SML power settings for CSC may vary between populations. Given China’s large population and the growing number of CSC patients receiving micropulse therapy, it is crucial to investigate SML parameters tailored to the Chinese population. Consequently, this study was designed to establish a cohort of 103 CSC patients in Shenzhen, China, to determine the lowest safe and effective SML power for treating acute and chronic CSC in this demographic.

## Methods

### Study design

This retrospective, nonrandom, comparative cohort clinical trial was conducted at the Shenzhen Eye Hospital Affiliated to Jinan University, Shenzhen, China. Institutional medical ethics committee approval was obtained (The Ethic number 2023KYPJ019). All interventions were conducted according to the tenets of the Declaration of Helsinki. All patients provided written informed consent to participate in this trial.

### Participants

The patients diagnosed with CSC based on clinical characteristics and findings on multimodal imaging were enrolled in this study. Criteria for inclusion were as follows: (1) 18 years old or older; (2) Acute CSC: disease duration was less than 4 months, FFA showed rapid accumulation of fluorescein under the neuroepithelium, and fluorescein leakage was ink diffusion type or smoke type (9). (3) Chronic CSC: disease duration greater than 6 months, FFA showed single or multiple focal, diffuse leakage of fluorescein in the macular area, with or without widespread RPE decompensation (9). Criteria for exclusion were: (1) patients who received PDT or laser treatment previously for CSC; (2) patients were diagnosed with other choroidal or retinal diseases such as CNV, diabetic retinopathy, and age-related macular degeneration; (3) patients who accepted ocular surgery during the study follow-up period.

All patients underwent ophthalmic examinations prior to the treatment, including best corrected visual acuity (BCVA) using LogMAR charts, dilated fundus examination, fundus photography, central macular thickness (CMT), and subretinal fluid (SRF) measured by spectral-domain optical coherence tomography (SD-OCT) (Cirrus HD OCT-5000, Zeiss, Jena, Germany), fundus fluorescein angiography (FFA), fundus auto-fluorescein (FAF) and indocyanine green angiography (ICGA) (Spectralis HRA, Heidelberg Engineering, Heidelberg, Germany). In the third month after treatment, patients were reassessed, and BCVA, fundus, and OCT examinations were performed. All treatments were performed by certified ophthalmologists with more than 20 years of experience.

### Treatment procedures

**Figure 1:**
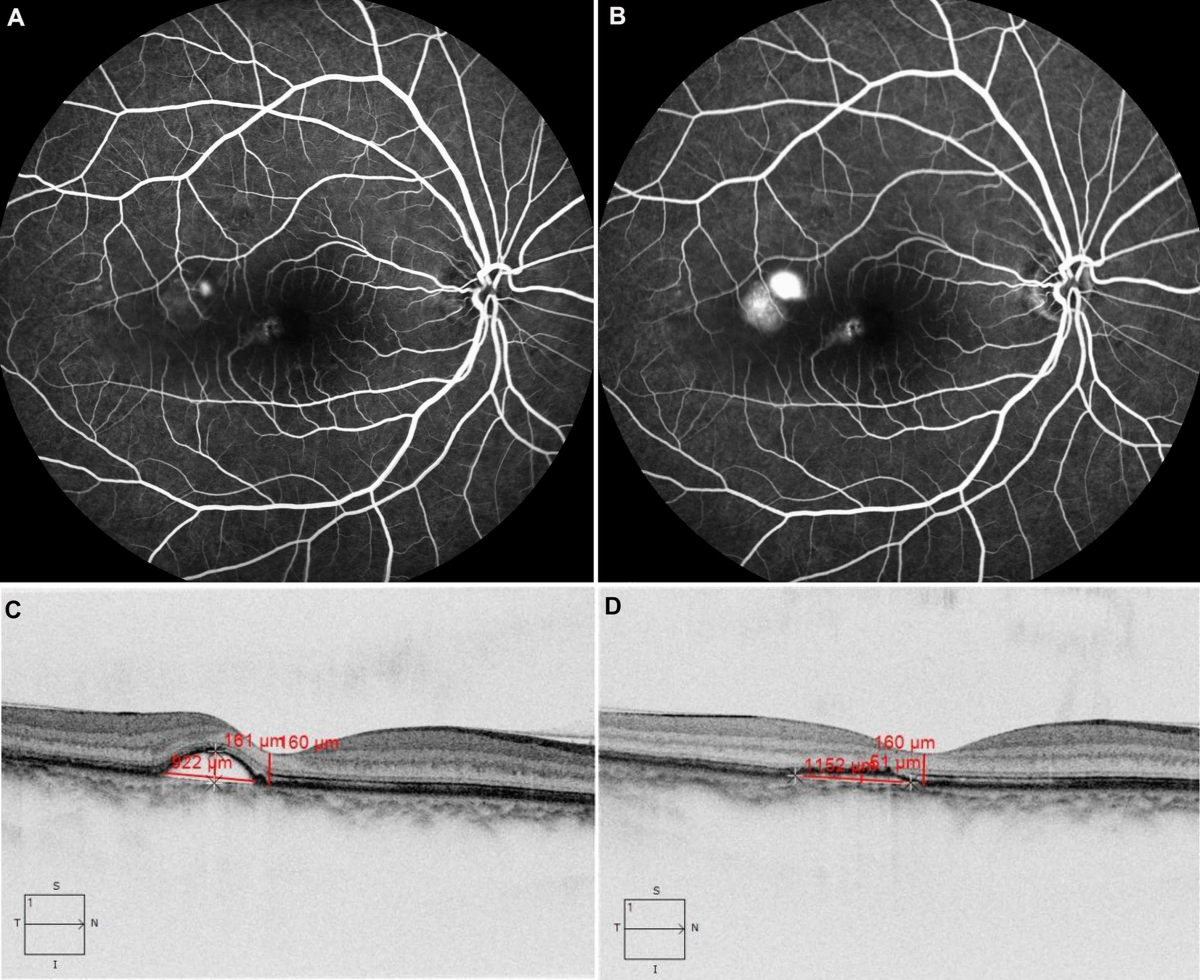
The patient with acute CSC received 577 nm yellow SML with 425 mW laser power. FFA revealed dye leakage located just above the macula in the early phase in panel **(A)** and subsequent pooling involving the macula in panel **(B)**. OCT showed retinal neuroepithelium detachment at the temporal side of the fovea **(C)**, after treatment, compared with that before treatment **(C)**. OCT showed subretinal fluid was absorbed completely at the third month **(D)**.

**Figure 2:**
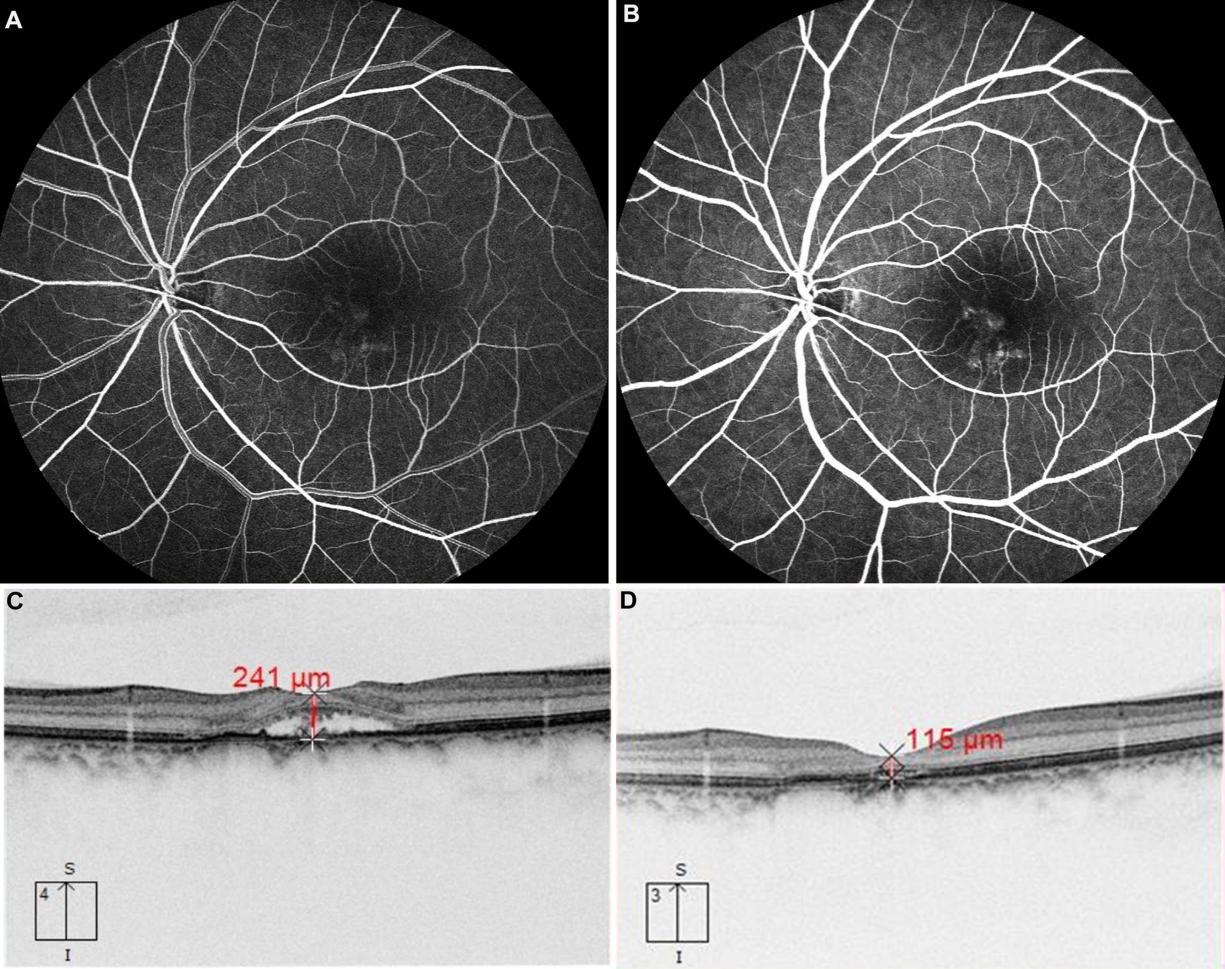
The patient with chronic CSC received 577 nm yellow SML with above 425 mW laser power. FFA revealed dye leakage located just below the macula in the early phase in panel **(A)** and subsequent pooling involving the macula in panel **(B)**. OCT showed retinal neuroepithelium detachment at the fovea **(C)**, after treatment, compared with that before treatment **(C)**. OCT showed subretinal fluid was absorbed completely at the third month **(D)**.

**Figure 3:**
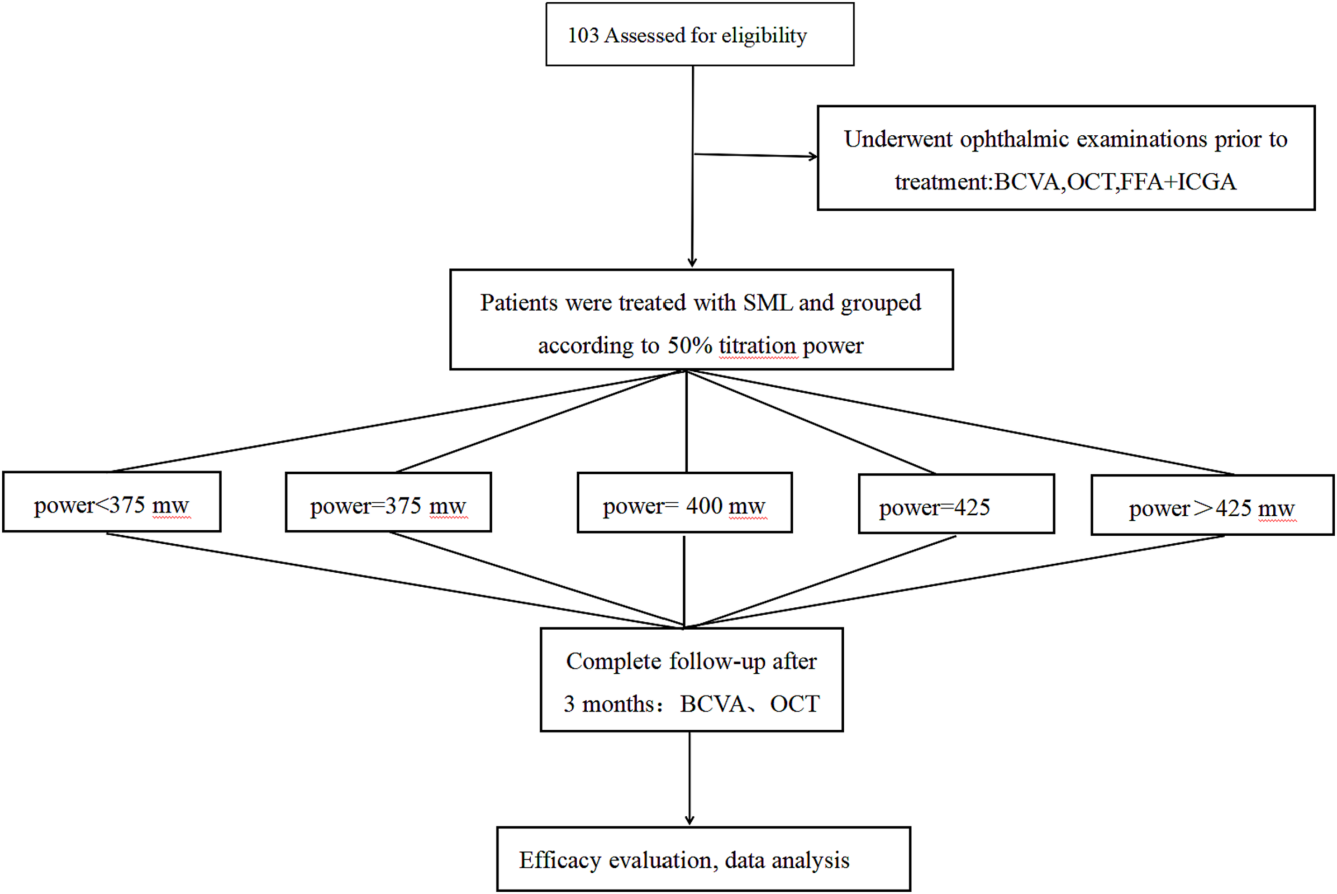
Study follow-up flowchart. SML, Subthreshold micropulse laser; BCVA, best-corrected visual acuity; OCT, optical coherence tomography; FFA, fundus fluorescein angiography; ICGA, indocyanine green angiography.

The SML treatment was performed with 577 nm yellow laser system (Supra Scan, Quantell Medical, Cedex, France) by a single retina specialist (QS Chen). The laser parameter was set to 5% duty cycle, and the exposure time was 0.2 s per spot. The spot sizes 140–160 μm were used, and the total laser burns were 100–800. The multi-spot model without spacing between the spots was chosen. The photocoagulation energy for each patient was titrated at the nasal upper quadrant of the retina in the mono-spot micropulse model. The power titration would gradually increase until a visible burn was seen, and then the power was reduced by 50% for individual treatment. Multimodal imaging of a CSC patient treated with SML is depicted in Figure 1 (acute CSC) and Figure 2 (chronic CSC). After finishing SML treatment, 127 eyes were divided into five treatment groups based on the titration power: (1) below 375 mW, (2) 375 mW, (3) 400 mW, (4) 425 mW, (5) above 425 mW. The follow-up flowchart is outlined in Figure 3.

### Outcome measurements

The primary outcomes included the change of BCVA and CMT from baseline to the third month. CMT was defined as the distance between the inner limiting membrane and the ellipsoid zone at the location with the maximal foveal dip on the SD-OCT scan (17). The secondary outcome included the complete absorption of SRF, defined as the CMT thinner than 220 μm in the third month.

### Statistical analyses

All analyses were performed using STATA statistical software (Stata V.14.0, Stata). BCVA was converted to the logarithm of the minimal angle of resolution (LogMAR) before statistical analyses. Data were presented as ‘mean ± standard deviation’. CMT and BVCA were used as continuous variables. SML power was used as a categorical variable. The linear regression model was used to assess the association of five different kinds of SML power with different factors and outcome indicators. A *p*-value less than 0.05 was considered statistically significant.

## Results

We enrolled 127 eyes from 103 CSC patients (76 male and 27 female) in Shenzhen Eye Hospital from December 2019 to December 2022. Sixty-one eyes (48.03%) were diagnosed with acute CSC, and 66 (51.97%) were chronic CSC. At baseline, the mean age of patients was 46.64 ± 9.71 years, the mean BCVA was 0.42 ± 0.38 logMAR, and the mean CMT was 372.09 ± 111.37 micrometers. During the three-month treatment follow-up period, For the treatments administered, the mean diameter of the SML laser beam ranged from 140 to 160 micrometers, with the mean number of laser points varying between 70 and 900. The average follow-up period after SML treatment was 3 months, and the baseline characteristics were balanced between five groups (all *p* > 0.05) (Supplementary Table 1). The baseline characteristics of patients in the study are shown in Table 1.

**Table 1:** Over all characteristic of participants at their first onset.

Variable	All participants	Male	Female	*p*
Number of patients	103	76	27	
Age (years)	46.64 (9.71, 30–72)	45.08 (8.88, 31–72)	51.04 (10.72, 30–70)	0.001
Number of eyes	127	94	33	
CMT at baseline (μm)	372.09 (111.37, 120–717)	377.73 (110.73, 120–717)	356.03 (113.31, 145–551)	0.874
CMT at follow-up (μm)	249.24 (98.20, 85–726)	252.31 (100.37, 85–726)	240.52 (92.68, 121–440)	0.589
Change of CMT (μm)	−122.52 (115.67, −413 to 233)	−124.79 (116.00, −413 to 233)	−116.06 (116.27, −367 to 128)	0.987
BCVA at baseline	0.42 (0.38, 0–2)	0.42 (0.41, 0–2)	0.42 (0.28, 0–1)	0.017
BCVA at follow-up	0.29 (0.36, −0.78 to 1.70)	0.28 (0.37, −0.78 to 1.70)	0.33 (0.33, 0.00–1.10)	0.413
Change of BCVA	−0.13 (0.26, −1.30 to 0.60)	−0.14 (0.28, −1.30 to 0.60)	−0.09 (0.18, −0.37 to 0.30)	0.006
Eye (*n*, %)				0.589
OD	68 (53.54%)	49 (52.13%)	19 (57.58%)	
OS	59 (46.46%)	45 (47.87%)	14 (42.42%)	
Type (*n*, %)				0.119
Acute	61 (48.03%)	49 (52.13%)	12 (36.36%)	
Chronic	66 (51.97%)	45 (48.39%)	21 (63.64%)	
Complete absorption of subretinal fluids				0.119
N	61 (48.03%)	49 (52.13%)	12 (36.36%)	
Y	66 (51.97%)	45 (47.87%)	21 (63.64%)	
Improvement on BCVA				0.611
N	47 (37.01%)	36 (38.30%)	11 (33.33%)	
Y	80 (62.99%)	58 (61.70%)	22 (66.67%)	
Improvement on CMT from OCT				0.547
N	19 (14.96%)	13 (13.83%)	6 (18.18%)	
Y	108 (85.04%)	81 (86.17%)	27 (81.82%)	

Continuous variables were displayed as: mean valve (standard deviation, range). CMT, central macular thickness; BCVA, best-corrected visual acuity (logMAR); OD, right eye; OS, left eye; N, no; Y, yes.

**Figure 4:**
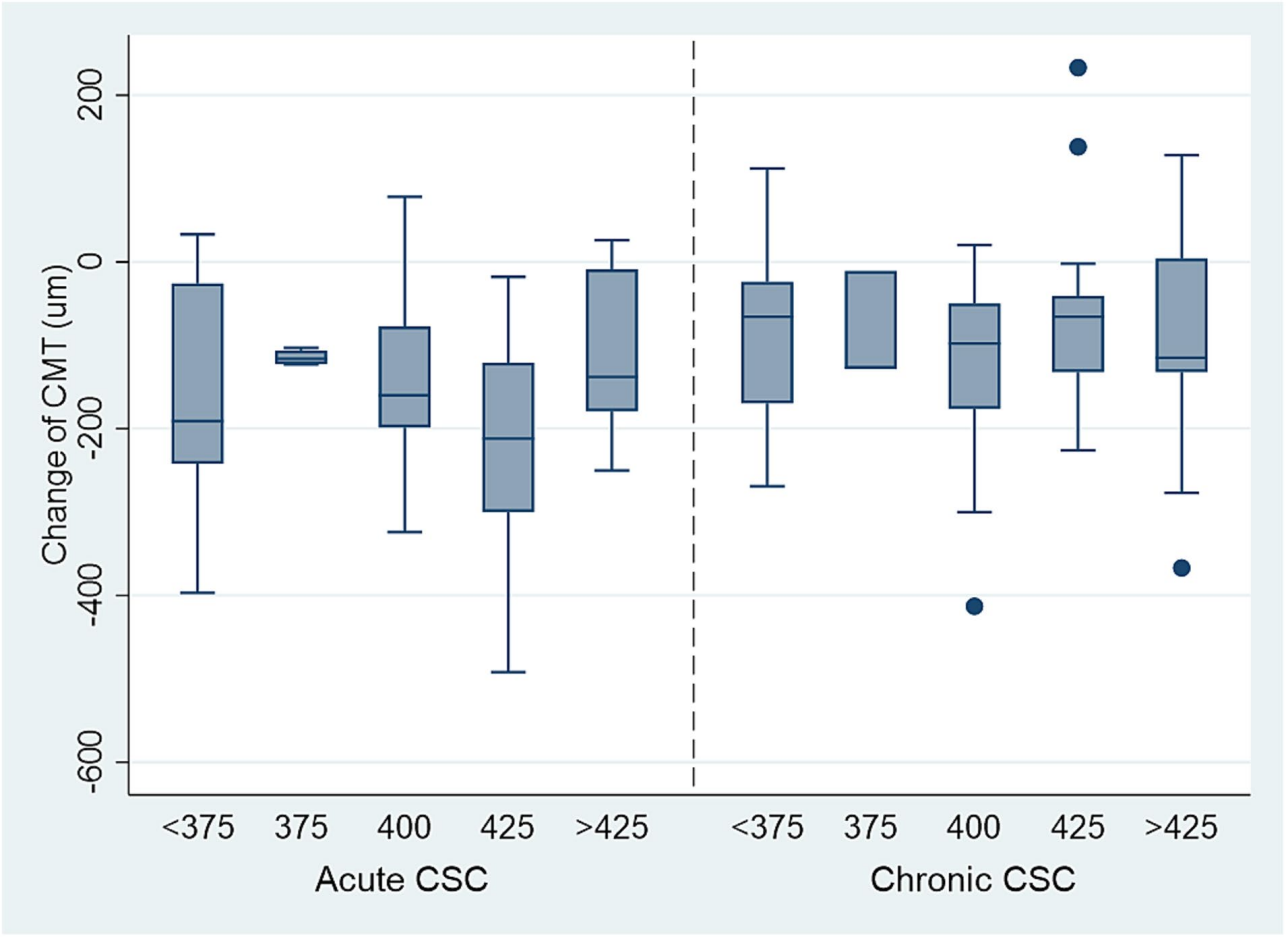
The decrease of CMT in acute and chronic CSC patients with different titration power (mw), 425 mW laser power is the safe, effective lowest power for treating acute CSC. The safe, effective lowest power for chronic CSC is above 425 mW.

The optimal laser power for acute CSC was determined to be 425 mW (mean ± SD: −225.50 ± 109.87 μm), which resulted in the most significant decrease in change of CMT (−225.50 μm vs. −171.24 μm vs. −114.50 μm vs. −130.54 μm vs. −68.00 μm, *p* < 0.001) (Table 2, Figure 4), but no significant increase in BCVA (−0.15 ± 0.10 logMAR vs. −0.20 ± 0.16 logMAR vs. −0.14 ± 0.11 logMAR vs. −0.17 ± 0.30 logMAR vs. −0.11 ± 0.14 logMAR, *p* > 0.05). For chronic CSC, the average change in CMT for each group was −93.91 ± 109.06 μm, −119.32 ± 105.56 μm, −88.67 ± 67.26 μm, −60.89 ± 106.86 μm, and −99.11 ± 157.32 μm (*p* = 0.04), respectively, the results showed that the change of CMT was significant in 375 mW (mean ± SD: −119.32 ± 105.56 μm, Table 3, Figure 4). The change of BCVA was similar trend (−0.54 ± 0.66 logMAR vs. −0.17 ± 0.23 logMAR vs. −0.10 ± 0.21 logMAR vs. −0.02 ± 0.30 logMAR vs. 0.05 ± 0.19 logMAR, *p* < 0.001) in Table 3.

**Table 2:** Ocular parameters with different titration power in patients with acute CSC.

Titration power	<375 mW	375 mW	400 mW	425 mW	>425 mW
Number of eyes	17	4	26	10	4
CMT at baseline (μm)	400.35 (129.11, 200–717)	313.25 (5.19, 310–321)	392.96 (102.08, 217–689)	423.90 (137.54, 226–608)	347.25 (129.64, 187–485)
CMT at follow-up (μm)	229.12 (81.45, 147–459)	198.75 (7.80, 189–208)	260.12 (83.94, 145–477)	198.40 (60.79, 130–345)	279.25 (119.42, 178–437)
Change of CMT (μm)	−171.24 (132.32, −397 to 33)	−114.50 (9.68, −123 to −103)	−130.54 (97.27, −324 to 78)	−225.50 (109.87, −384 to −66)	−68.00 (93.81, −179 to 26)
BCVA at baseline	0.30 (0.27, 0–0.82)	0.15 (0.13, 0.05–0.30)	0.33 (0.41, 0.00–2.00)	0.32 (0.19, 0.10–0.70)	0.35 (0.26, 0.10–0.70)
BCVA at follow-up	0.10 (0.14, 0.00–0.40)	0.01 (0.02, 0.00–0.05)	0.16 (0.17, 0.00–0.70)	0.17 (0.18, 0.00–0.52)	0.25 (0.17, 0.10–0.40)
Change of BCVA	−0.20 (0.16, −0.43 to 0.10)	−0.14 (0.11, −0.26 to −0.05)	−0.17 (0.30, −1.30 to 0.22)	−0.15 (0.10, −0.20 to 0.07)	−0.11 (0.14, −0.30 to 0.00)
Eye (*n*, %)					
OD	10 (58.82%)	2 (50.00%)	14 (53.85%)	7 (70.00%)	2 (50.00%)
OS	7 (41.18%)	2 (50.00%)	12 (46.15%)	3 (30.00%)	2 (50.00%)
Complete absorption of subretinal fluids					
N	7 (41.18%)	0 (0.00%)	17 (65.38%)	3 (30.00%)	2 (50.00%)
Y	10 (58.82%)	4 (100.00%)	9 (34.62%)	7 (70.00%)	2 (50.00%)
Improvement on BCVA					
N	4 (23.53%)	0 (0.00%)	9 (34.62%)	4 (40.00%)	2 (50.00%)
Y	13 (76.47%)	4 (100.00%)	17 (65.38%)	6 (60.00%)	2 (50.00%)
Improvement on CMT from OCT					
N	2 (11.76%)	0 (0.00%)	4 (15.38%)	0 (0.00%)	2 (50.00%)
Y	15 (88.24%)	4 (100.00%)	22 (84.62%)	10 (100.00%)	2 (50.00%)

Continuous variables were displayed as: mean valve (standard deviation, range). CMT, central macular thickness; BCVA, best-corrected visual acuity (logMAR); OD, right eye; OS, left eye; N, no; Y, yes.

**Table 3:** Ocular parameters with different titration power in patients with chronic CSC.

Titration power	<375 mW	375 mW	400 mW	425 mW	>425 mW
Number of eyes	11	3	25	18	9
CMT at baseline (μm)	339.55 (109.30, 180–494)	285.00 (65.82, 211–337)	345.48 (118.10, 120–599)	380.44 (89.51, 202–507)	364.11 (108.23, 248–546)
CMT at follow-up (μm)	245.64 (103.08, 142–430)	196.33 (15.82, 179–210)	226.88 (102.99, 85–440)	319.56 (125.82, 200–726)	265.00 (89.63, 135–400)
Change of CMT (μm)	−93.91 (109.06, −269 to 112)	−119.32 (105.56, −413 to 20)	−88.67 (67.26, −128 to 11)	−60.89 (106.86, −226 to 233)	−99.11 (157.32, −367 to 128)
BCVA at baseline	0.54 (0.37, 0–1.15)	0.28 (0.22, 0.09–0.52)	0.61 (0.45, 0–1.70)	0.45 (0.40, 0–1.30)	0.44 (0.36, 0–1.00)
BCVA at follow-up	0.37 (0.34, 0.00–0.92)	−0.26 (0.45, −0.78 to 0.00)	0.51 (0.41, 0.00–1.40)	0.44 (0.44, 0.00–1.70)	0.49 (0.42, 0.10–1.10)
Change of BCVA	−0.17 (0.23, −0.80 to 0.00)	−0.54 (0.66, −1.30 to 0.10)	−0.10 (0.21, −0.48 to 0.46)	−0.01 (0.30, −0.48 to 0.60)	0.05 (0.19, −0.22 to 0.27)
Eye (*n*, %)					
OD	5 (45.45%)	1 (33.33%)	14 (56.00%)	8 (44.44%)	5 (55.56%)
OS	6 (54.55%)	2 (66.67%)	11 (44.00%)	10 (55.56%)	4 (44.44%)
Complete absorption of subretinal fluids					
N	5 (45.45%)	0 (0.00%)	7 (28.00%)	14 (77.78%)	6 (66.67%)
Y	6 (54.55%)	3 (100.00%)	18 (72.00%)	4 (22.22%)	3 (33.33%)
Improvement on BCVA					
N	4 (36.36%)	0 (0.00%)	9 (36.00%)	9 (50.00%)	6 (66.67%)
Y	7 (63.64%)	3 (100.00%)	16 (64.00%)	9 (50.00%)	3 (33.33%)
Improvement on CMT from OCT					
N	2 (18.18%)	0 (0.00%)	3 (12.00%)	3 (16.67%)	3 (33.33%)
Y	9 (81.82%)	3 (100.00%)	22 (88.00%)	15 (83.33%)	6 (66.67%)

Continuous variables were displayed as: mean valve (standard deviation, range). CMT, central macular thickness; BCVA, best-corrected visual acuity (logMAR); OD, right eye; OS, left eye; N, no; Y, yes. * Statistically significant.

The line regression analyses showed the more significant association between baseline age and titration power for chronic CSC patients (coef: 1.14; 95%CI: 0.10–2.18; *p* = 0.032) in Supplementary Table 2. Furthermore, the results of line regression suggested that the change of BCVA with chronic CSC patients was obviously significant after 375 mW SML treatment (coef: −0.52; 95%CI: −0.89 to −0.15; *p* = 0.007). However, for acute CSC, the changes in CMT were significantly associated with changes in BCVA (coef: 0.00; 95%CI: 0.00–0.00; *p* = 0.016) in Table 4.

**Table 4:** The association between the change of BCVA and ocular characteristics.

Variable	Acute CSC		Chronic CSC	
	*p*	coef (95%CI)	*p*	coef (95%CI)
Gender	0.921	0.01 (−0.15, 0.17)	0.687	0.03 (−0.13, 0.19)
Age	0.548	0.00 (−0.01, 0.00)	0.158	−0.01 (−0.01, 0.00)
CMT at baseline	0.363	0.00 (0.00, 0.00)	0.631	0.00 (0.00, 0.00)
Change of CMT	0.016*	0.00 (0.00, 0.00)	0.208	0.00 (0.00, 0.00)
Titration power				
<375 mW	0.966	−0.01 (−0.29, 0.27)	0.057	−0.25 (−0.51, 0.01)
375 mW	0.940	0.01 (−0.34, 0.37)	0.007*	−0.52 (−0.89, −0.15)
400 mW	0.854	−0.02 (−0.29, 0.24)	0.342	−0.11 (−0.32, 0.11)
425 mW	0.541	0.09 (−0.21, 0.40)	0.454	−0.08 (−0.31, 0.14)
>425 mW	–	Ref.	–	Ref.
Laser beam diameter	0.670	0.00 (0.00, 0.00)	0.300	0.00 (0.00, 0.00)
Number of laser points	0.984	0.00 (0.00, 0.00)	0.459	0.00 (0.00, 0.00)

CSC, Central serous chorioretinopathy; CMT, central macular thickness; BCVA, best-corrected visual acuity (logMAR). The linear regression model was adjusted by the variables in the table. *Statistically significant.

In Table 5, we found that the change in CMT was significantly associated with CMT at baseline for acute CSC patients (coef: −0.71; 95%CI: −0.88 to −0.53; *p* = 0.010). In contrast, for chronic CSC, no significant correlation was found between changes in CMT and baseline characteristics (all *p* > 0.05). The improvement in CMT was most significant in the 375-mw group (coef: −0.52; 95%CI: −0.89 to −0.15; *p* = 0.007).

**Table 5:** The association of change of CMT with ocular characteristics.

Variable	Acute CSC		Chronic CSC	
	*p*	coef (95%CI)	*p*	coef (95%CI)
Gender	0.141	−36.46 (−85.46, 12.54)	0.687	0.03 (−0.13, 0.19)
Age	0.970	−0.04 (−2.29, 2.20)	0.158	−0.01 (−0.01, 0.00)
CMT at baseline	0.010*	−0.71 (−0.88, −0.53)	0.631	0.00 (0.00, 0.00)
Titration power				
<375 mW	0.046	−86.81 (−171.97, −1.64)	0.057	−0.25 (−0.51, 0.01)
375 mW	0.076	−97.41 (−205.53, 10.70)	0.007*	−0.52 (−0.89, −0.15)
400 mW	0.223	−51.40 (−135.15, 32.34)	0.342	−0.11 (−0.32, 0.11)
425 mW	0.010	−122.20 (−213.58, −30.81)	0.454	−0.08 (−0.31, 0.14)
>425 mW	–	Ref.	–	Ref.
Laser beam diameter	0.272	0.26 (−0.21, 0.72)	0.300	0.00 (0.00, 0.00)
Number of laser points	0.426	−0.06 (−0.23, 0.10)	0.459	0.00 (0.00, 0.00)

CSC, Central serous chorioretinopathy; CMT, central macular thickness. The linear regression model was adjusted by the variables in the table. *Statistically significant.

## Discussion

Given the self-limiting nature of CSC, previous guidelines recommended monitoring patients for at least 3 months before initiating treatment. However, emerging evidence suggests that early intervention may lead to better visual outcomes than waiting for spontaneous resolution (18–20). Prompt treatment has been shown to enhance anatomical recovery and improve long-term visual outcomes (21). Various treatment modalities for CSC have been explored (22, 23), and SML has become an effective choice. Our study aimed to determine the safe lowest effective power of 577-nm yellow SML needed to treat acute and chronic CSC. We found that SML at 425 mW was a safe and effective low-power option for acute CSC, and 375 mW was optimal for chronic CSC. Meanwhile, our results showed a positive correlation between age and the required SML power for chronic CSC. The chronic CSC patients need more higher power as they get older. As far as we know, our study was the first clinical research in China that explored different SML power settings for acute and chronic CSC.

Several previous studies reported that different optimal laser parameters had profound effects on acute and chronic CSC using SML treatment (11, 24, 25). Numerous researches have predominantly examined aspects such as laser threshold percentage, wavelength, and duty cycle. For example, Lijun Zhou investigated different percentage settings for acute central serous chorioretinopathy (26). Alfred K. Yu evaluated the impact of various wavelengths on the retina through histological and differential protein expression studies (27). Jenny Wang assessed the pros and cons of varying temporal modulation strategies (28). Nonetheless, a report suggested that excessive laser power with SML might cause retinal damage and a retinal shock response despite precise titration procedures (29). Therefore, it is necessary to investigate the optimal and safe laser power settings.

The 577 nm wavelength falls within the yellow spectral range, allowing it to penetrate the lens and superficial hemorrhage while avoiding absorption by lutein, thus minimizing phototoxic effects (30). Research indicates that 577 nm laser treatment is associated with a lower risk of Bruch’s membrane rupture (31). A recent retrospective study also demonstrated that 577 nm SML can achieve outcomes comparable to conventional laser treatments without causing RPE damage (32). In our study, we treated CSC using a 577 nm yellow SML. We tested power levels ranging from 160 mW to 475 mW, divided into five groups: <375 mW, 375 mW, 400 mW, 425 mW, and >425 mW, and compared the treatment outcomes. Furthermore, our findings implied that a laser power of 425 mW was more effective than lower power levels in reducing CMT, improving BCVA, and increasing the rate of SRF absorption in cases of acute CSC. Based on these results, we considered that power levels below 425 mW may be insufficient for restoring macular and visual function, in comparison, power levels above 425 mW could lead to more significant retinal damage. Therefore, 425 mW appears to be the optimal laser power for treating acute CSC, identifying the best balance between efficacy and minimizing potential damage.

Although the etiology of CSC remains unknown, it mainly affects middle-aged men and is remarkably associated with corticosteroid use, stress, and differences in ethnic and sociodemographics (33–35). In CSC, the function of RPE is mainly to maintain the outer blood-retina barrier, which was damaged due to congestion, hyper-permeability, and thickening of the choroid. The resulting loss of integrity in this barrier leads to the accumulation of SRF, which affects photoreceptor function and leads to a decrease in vision (3, 36). SML treatment may significantly improve the function of vision, which may be attributed to the fact that SML targets the RPE and further promotes SRF resolution (37). Besides, melanin in the RPE absorbs the photons’ energy, which is dissipated in the form of heat (38). This SML treatment was able to increase the expression of heat shock proteins and restore cellular function in the RPE (12). Therefore, it can be particularly relevant for treating chorioretinal diseases such as CSC. With SML treatment, the aim is to selectively target the RPE without causing visible tissue damage.

Acute CSC is characterized by local SRF accumulation in the macular area, usually without RPE detachment (12). The characteristic of chronic CSC is persistent serous neuroretinal detachment, which can be small or wide in size and multifocal in multiple leakage areas (39). A common imaging feature of atrophic RPE changes on FFA, ranging from a single localized area to extensively DARA (diffuse atrophic RPE alterations). As the disease progresses, the melanin in RPE cells gradually decreases. In this study, chronic CSC patients treated with 375 mW laser energy showed better functional outcomes, including improved BCVA, reduced CMT, and a higher rate of SRF absorption. This suggests that the optimal laser power for chronic CSC is lower than that for acute CSC, possibly due to the disease’s longer duration and the increased choroidal permeability in chronic cases. A key factor is that the SML selectively targets the RPE layer, stimulating RPE cells for repair (40, 41). The therapeutic effect of SML is enhanced when the RPE layer is thicker. However, in chronic CSC, SRF persists for 4 to 6 months, worsening RPE cell damage, leading to RPE decompensation and atrophy, which reduces the thickness of the RPE layer (42). Besides, because of the RPE damage of chronic CSC, the melanin in RPE cells gradually decreased, and fewer normal RPE cells can absorb laser energy. Therefore, chronic CSC requires lower laser energy to reduce the retinal damage caused by SML.

There are several limitations to this study. The primary limitation is the relatively short follow-up period of only three months. Since recurrences are common in CSC, this short duration may limit our ability to assess the long-term efficacy of different laser power settings. Additionally, all patients were recruited from a single institution, which could introduce selection bias. Lastly, the underlying mechanisms of how different laser power affects CSC remain unclear and warrant further investigation.

## Conclusion

In conclusion, the current study suggested that 425 mW and 375 mW are the safe lowest effective power to treat acute and chronic CSC, respectively, which may provide the most optimal and safe protocol for achieving anatomical and functional improvements in CSC. It could improve clinicians to make better clinical management for CSC patients. However, these results should be confirmed by large and well-designed studies to illustrate the specific mechanism.

## Data Availability

The raw data supporting the conclusions of this article will be made available by the authors without undue reservation.
