# Supplementary material for: The safe lowest effective power of subthreshold micropulse laser treatment in Chinese patients with acute or chronic central serous chorioretinopathy
Source: Front Med (Lausanne). 2024 Nov 6;11:1494402. doi: 10.3389/fmed.2024.1494402 (PMC11576171; doi:10.3389/fmed.2024.1494402)
Supplement: Supplementary file 1 [file Table_1.DOCX]

Supplement Table 1 Ocular parameters with different titration power.

| Titration power | <375 mw | 375 mw | 400 mw | 425 mw | >425 mw | *P* |
| --- | --- | --- | --- | --- | --- | --- |
| Number of eyes | 28 | 7 | 51 | 28 | 13 |  |
| CMT at baseline (um) | 376.46 (123.36, 180~717) | 301.14 (41.05, 211~337) | 369.69 (111.71, 120~689) | 395.96 (108.63, 202~608) | 358.92 (109.90, 187~546) | 0.361 |
| CMT at follow-up (um) | 235.61 (89.08, 142~459) | 197.71 (10.75, 179~210) | 243.82 (94.32, 85~477) | 276.29 (121.22, 130~726) | 269.38 (94.70, 135~437) | 0.260 |
| Change of CMT (um) | -140.86 (127.51, -397~112) | -103.43 (41.78, -128~-11) | -125.04 (100.56, -413~78) | -119.68 (132.91, -384~233) | -89.54 (137.56, -367~128) | 0.743 |
| BCVA at baseline | 0.39 (0.33, 0~1.15) | 0.21 (0.17, 0.046~0.52) | 0.47 (0.45, 0~2) | 0.40 (0.34, 0~1.30103) | 0.42 (0.32, 0~1) | 0.528 |
| BCVA at  follow-up | 0.21 (0.27, 0.00~0.92) | -0.10 (0.30,  -0.78~0.05) | 0.33 (0.36, 0.00~1.40) | 0.34 (0.39, 0.00~1.70) | 0.42 (0.37, 0.10~1.10) | 0.010 |
| Change of BCVA | -0.19 (0.19,  -0.80~0.10) | -0.31 (0.44,  -1.30~-0.05) | -0.14 (0.26,  -1.30~0.46) | -0.06 (0.26,  -0.48~0.60) | 0.00 (0.18,  -0.30~0.27) | 0.034 |
| Eye (n, %) |  |  |  |  |  | 0.986 |
| OD | 15 (53.57%) | 3 (42.86%) | 28 (54.90%) | 15 (53.57%) | 7 (53.85%) |  |
| OS | 13 (46.43%) | 4 (57.14%) | 23 (45.10%) | 13 (46.43%) | 6 (46.15%) |  |
| Type (n, %) |  |  |  |  |  | 0.243 |
| Acute | 17 (60.71%) | 4 (57.14%) | 26 (50.98%) | 10 (35.71%) | 4 (30.77%) |  |
| Chronic | 11 (39.29%) | 3 (42.86%) | 25 (49.02%) | 18 (64.29%) | 9 (69.23%) |  |
| Complete absorption of subretinal fluids | | | |  |  | 0.049 |
| N | 12 (42.86%) | 0 (0.00%) | 24 (47.06%) | 17 (60.71%) | 8 (61.54%) |  |
| Y | 16 (57.14%) | 7 (100.00%) | 27 (52.94%) | 11 (39.29%) | 5 (38.46%) |  |
| Improvement on BCVA | | | |  |  | 0.051 |
| N | 8 (28.57%) | 0 (0.00%) | 18 (35.29%) | 13 (46.43%) | 8 (61.54%) |  |
| Y | 20 (71.43%) | 7 (100.00%) | 33 (64.71%) | 15 (53.57%) | 5 (38.46%) |  |
| Improvement on CMT from OCT | | | |  |  | 0.119 |
| N | 4 (14.29%) | 0 (0.00%) | 7 (13.73%) | 3 (10.71%) | 5 (38.46%) |  |
| Y | 24 (85.71%) | 7 (100.00%) | 44 (86.27%) | 25 (89.29%) | 8 (61.54%) |  |

Footnotes: Continuous variables were displayed as: mean valve (standard deviation, range). CMT: central macular thickness. BCVA: best-corrected visual acuity (logMAR). OD: right eye. OS: left eye. N: no. Y: yes.

Supplement Table 2. The association of titration power with baseline characters of participants.

|  | Acute CSC | | Chronic CSC | |
| --- | --- | --- | --- | --- |
| Variable | *P* | coef (95%CI) | *P* | coef (95%CI) |
| Gender | 0.766 | 5.45 (-31.05, 41.94) | 0.214 | 12.54 (-7.41, 32.48) |
| Age | 0.521 | 0.55 (-1.16, 2.26) | 0.032* | 1.14 (0.10, 2.18) |
| BCVA at baseline | 0.604 | 12.41 (-35.19, 60.00) | 0.383 | -10.48 (-34.34, 13.37) |
| CMT at baseline | 0.269 | -0.07 (-0.20, 0.06) | 0.324 | 0.04 (-0.04, 0.13) |

Footnotes: CSC: central serous chorioretinopathy. CMT: central macular thickness. BCVA: best-corrected visual acuity (logMAR). The linear regression model was adjusted by the variables in the table. * Statistically Significant.
